# Natural Extracts and Their Applications in Polymer-Based Active Packaging: A Review

**DOI:** 10.3390/polym16050625

**Published:** 2024-02-25

**Authors:** Jiawei Li, Hui Sun, Yunxuan Weng

**Affiliations:** 1 College of Light Industry Science and Engineering, Beijing Technology and Business University, Beijing 100048, China; ivy_li0828@163.com; 2 Beijing Key Laboratory of Quality Evaluation Technology for Hygiene and Safety of Plastics, Beijing Technology and Business University, Beijing 100048, China

**Keywords:** natural preservative, active packaging, food preservation

## Abstract

At a time when food safety awareness is increasing, attention is paid not only to food and additives but also to packaging materials. Most current food packaging is usually made of traditional petroleum-based polymeric materials, which are not biodegradable and have adverse effects on the environment and health. In this context, the development of new non-toxic and biodegradable materials for extending the best-before date of food is receiving increasing attention. In addition, additives in packaging materials may migrate outward, resulting in contact with food. For this reason, additives are also seen as a transition from synthetic additives to natural extracts. Active extracts from animals and plants having good antioxidant and antibacterial properties are also beneficial for human health. It is indisputable that active extracts are ideal substitutes for synthetic additives. Polymer packaging materials combined with active extracts not only maintain their original mechanical and optical properties and thermal stability but also endow polymers with new functions to extend the shelf life of food. This review paper provides an overview of this promising natural extract-containing polymer-based active packaging, with a focus on plant essential oils (containing phenolics, monoterpenes, terpene alcohols, terpene ketones, and aldehydes), pigments (procyanidins), vitamins (vitamin B), and peptides (nisin). In particular, this paper covers the research progress of such active extracts, in single or compound forms, combined with diverse polymers (mostly biopolymers) for food packaging applications with particular focus on the antioxidant and antibacterial properties of packaging materials.

## 1. Introduction

More and more people are paying attention to the proportion of fresh fruits and vegetables in their daily diets, shifting toward a healthier lifestyle. It is noteworthy that fresh fruits and vegetables are vectors for bacteria (*Escherichia coli*, *Staphylococcus aureus*, *Listeria monocytogenes*, *Salmonella*, *Bacillus*, etc.), fungi (spoilage yeast, *Aspergillus niger*, *Penicillium*, etc.), and viruses (norovirus, etc.) spreading to consumers. Thus, the behavior of omophagia will increase the global burden of foodborne diseases.

To prevent foodborne diseases, food must be prepared, stored, and transported scientifically. In the process of continuous evolution and elimination, more drug-resistant bacteria have survived [1], causing a greater chance of contamination by bacterial pathogens before reaching consumers. Antibiotics, as drugs, are generally not recommended to be directly added to or in contact with food, which could affect consumers’ sensory experience [2]. Therefore, it is increasingly important to develop more up-to-date preservation methods.

**Figure 1:**
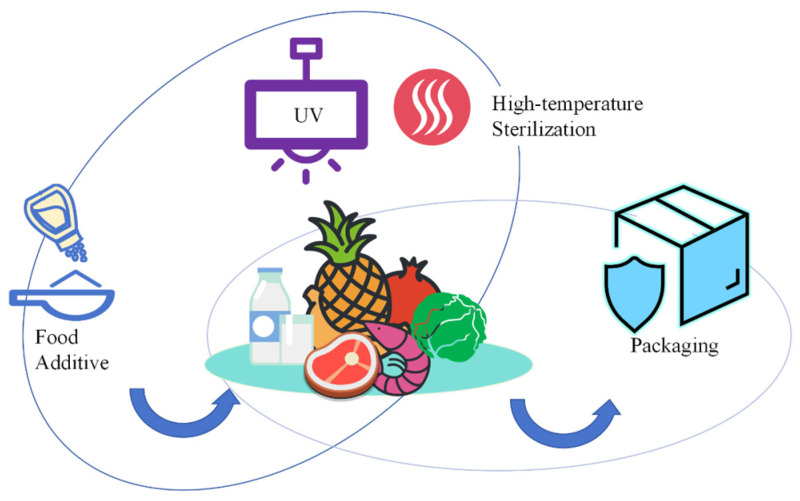
Preservation methods for food.

Previously, food preservation was mainly considered from both external and internal perspectives, as shown in Figure 1. The external perspective is generally to use cling wrap films or vacuum packaging. This solution has a limited scope of use and a short shelf life. In the post-pandemic era, consumers are paying more attention to the antibacterial properties of packaging materials. Bio-based materials or biopolymers (chitosan, polylactic acid (PLA), cellulose, sodium alginate, etc.) are becoming the preferred choices for developing new packaging materials, especially for food packaging, owing to their environmentally friendly, efficient utilization of natural resources, and non-toxic properties.

The internal perspective generally focuses on the food itself, which includes pickling, high-temperature steaming, UV disinfection, and the direct addition of food-grade preservatives. The former is prone to a decrease in sensory experience. As for the latter, most of the food preservatives [3,4,5] are chemically synthesized, while a few, such as natamycin [6] and nisin [7], are natural. Preservatives can maintain the freshness of food but cannot inhibit or kill microorganisms. So, the momentum in preservation requirements for raw meat, fresh-cut fruits, vegetables, and seafood is diminishing.

The research and development of natural preservative agents is gradually becoming a hot topic of central focus. Natural preservative extracts are ideal substitutes for synthetic food preservatives because they come from nature, are widely distributed, have large reserves, and are green and healthy. This review introduces the research progress of active packaging (AP) containing natural active extracts in food preservation, including antioxidant and antimicrobial properties, and the main and common natural active extracts, including molecular structures, are classified and summarized.

## 2. Antibacterial and Antioxidant Properties of Natural Extracts

Three common active chemical components (i.e., phenolics, terpenes, and aldehydes) are present in plants. These organic compounds will be introduced together with their food preservation applications below.

### 2.1. Phenolics

**Figure 2:**
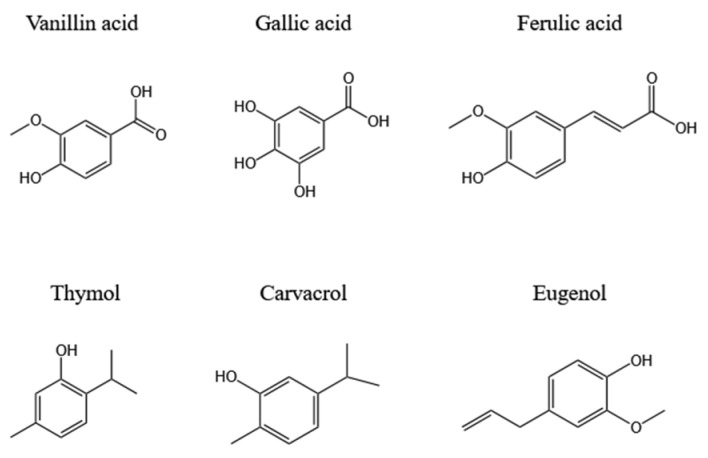
Phenolic compounds in plants.

Many studies have shown that phenolic compounds in plants, such as vanillin, gallic acid, eugenol, etc., have universal antioxidant properties (the free radical scavenging effect); therefore, they have potential value in food preservation applications. The common phenolic compounds related to antioxidant and antimicrobial properties are shown in Figure 2.

Kimani [8] tested the antifungal properties of two phenols and three synthetic preservatives (sodium benzoate, potassium sorbate, and sodium diacetate). The results showed that compared with the phenolic–synthetic combinations, the vanillin–cinnamic acid combination had higher activity against spoilage yeast adhesion on abiotic surfaces, biofilm formation, and planktonic growth.

Yao [9] studied the inhibition of eugenol against the biosynthesis of ochratoxin A (OTA) and the transcription of growth of *Aspergillus carbonarius* (*A. Carbonarius*). OTA is the most common fungal toxin in grapes and their varieties and is mainly composed of anthraquinone. Consequently, it was possible to use eugenol to preserve some fruits.

Pradhan [10] studied the chemical compounds and antioxidant properties of MeOH extracts from different plant parts of *Inula grandiflora*. According to the results from High Performance Liquid Chromatography–Photodiode Array Detection (HPLC–PDA), most of the bioactive polyphenol compounds were in roots. Extracts from roots also had the highest antioxidant potential.

### 2.2. Terpenes

#### 2.2.1. Monoterpene Alcohol

**Figure 3:**
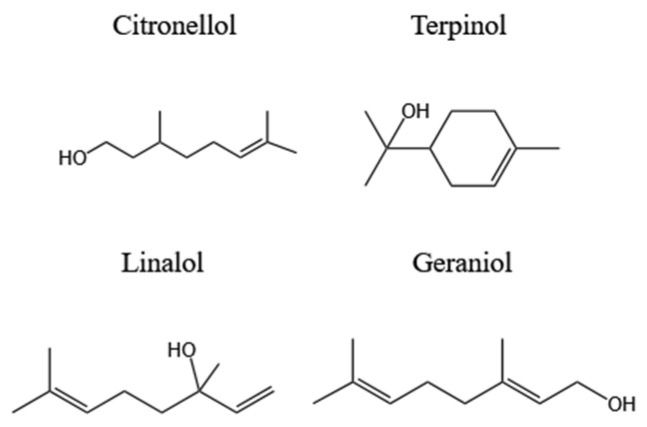
Monoterpene alcohols in plants.

Monoterpene alcohols can enhance the immune system’s resistance and thereby resist infection. Common monoterpene alcohol compounds related to antioxidant and antimicrobial properties are shown in Figure 3.

Silva [11] proved that the inhibitory effects of the two isomers of β-citronellol on two types of *Candida* (*C. albicans* and *C. tropicalis*) are similar. β-citronellol impacted fungi’s cell membrane but not the wall.

#### 2.2.2. Monoterpene

**Figure 4:**
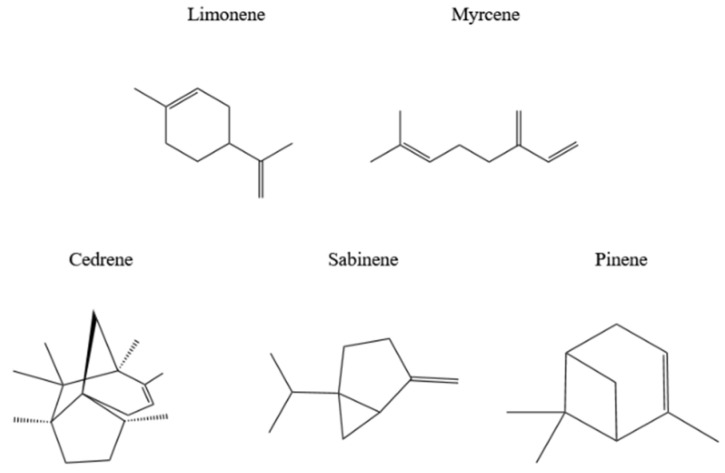
Monoterpenes in plants.

A terpene is a kind of substance commonly found in plant extracts and contains monoterpenes (such as limonene, myrcene, and cedrene), sesquiterpenes (such as copaene, β-caryophyllene), and so on. Common monoterpene compounds related to antioxidant and antimicrobial properties are shown in Figure 4.

Wang [12] encapsulated d-limonene lipid carriers (NLCs) with candelilla wax as organo-gelators and pea protein isolate (PPI) nanoparticles as emulsifiers. NLCs showed high physical stability, a good inhibition effect on the growth of *Botrytis cinerea*, and can effectively retain d-limonene. Furthermore, in vitro and in vivo studies were conducted, and the results showed that the degree of decay of tomatoes soaked by NLCs obviously decreased. The surfaces of the soaked tomatoes were brighter and smoother.

**Figure 5:**
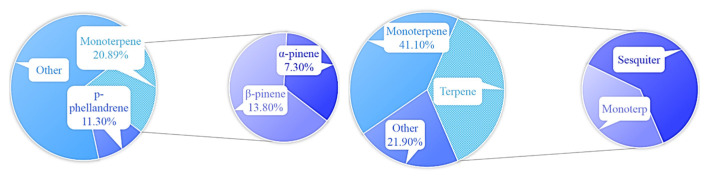
Main chemical compounds from different parts of ginger.

Huong [13] studied the potential compounds of the rhizome essential oil of ginger, whose main chemical compounds were different from parts of plants. The main chemical compounds in ginger essential oils are shown in Figure 5.

### 2.3. Ketones and Aldehydes

**Figure 6:**
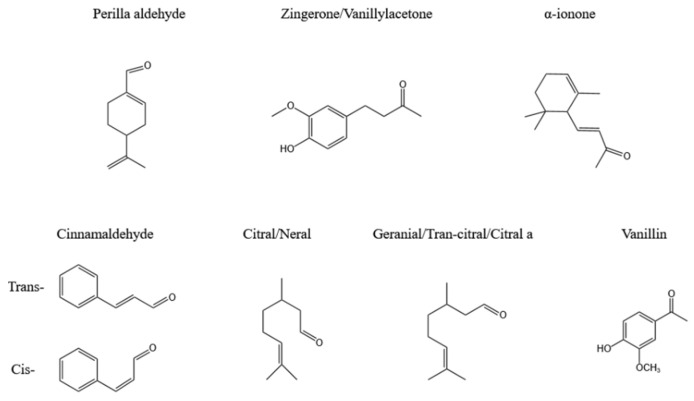
Terpene ketones and aldehydes in plants.

Ketones and aldehydes are commonly found in plants including ionone, zingerone, cinnamaldehyde, citral, and perillaldehyde, as shown in Figure 6.

Cinnamaldehyde is a broad-spectrum antibacterial agent that is widely present in camphor plants and is divided into two types: trans and cis. Generally speaking, the content of trans is much higher than that of cis.

**Figure 7:**
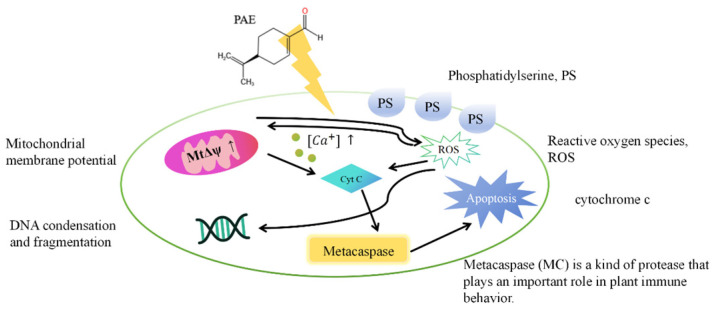
Antibacterial mechanism diagram of PEA.

Akbari [14] investigated the activity of antibiofilms against pathogenic bacteria through subcritical water and aqueous-ethanolic extracts of ginger in broth cultures and food model agents. The results showed that 50% (*v/v*) ginger subcritical water extract had the highest antibiofilm activity, similar to 0.5% peracetic acid (control). Tian [15] found that perillaldehyde (PEA) could induce *Aspergillus flavus* (fungal) apoptosis, as shown in Figure 7, providing a new mechanism for exploring a possible antifungal agent for food preservation.

### 2.4. The Preservative Properties of Mixtures of Natural Extracts

Mugahi [16] investigated the effect of extracts of turmeric, cinnamon, and lemon on the shelf life, nutrients (moisture, protein, and fat), and oxidative spoilage (peroxide value, PV, free fatty acid, FFA, and acidity rate) of carp fish in cold storage at 4 °C. The results showed that soaking carp slices with plant extracts significantly reduced the decay index, maintained the nutritional value of the fish, increased the protein content, and reduced fat and ash contents. In short, the shelf life was increased. Byun [17] collected four citrus extracts from lemon, yuzu, naringin, and resveratrol. During storage, the effect of using antibacterial spray to control *Salmonella* on whole and fresh-cut cucumbers at different temperatures was investigated. Results suggested that fruit extracts, especially lemon, exerted effective anti-*Salmonella* efficacy on the whole cucumbers and subsequently reduced the cross contamination of this pathogen during the fresh-cut process. A kind of bacterium called *Pectobacterium carotovorum* subsp. *Carotovorum* (Pcc) can damage Chinese cabbage. Cai [18] extracted the essential oil of *Polygonum orientale* L. (POEO) and studied its 29 chemical components (β-ionone, phytol, etc.), which had potential application prospects for controlling this bacterium. POEO increased the surface potential, increased hydrophobicity, damaged cell walls, destroyed the integrity and permeability of cell membranes, reduced membrane potential, and changed membrane protein conformation. POEO inhibited the activities of pyruvate kinase, succinate dehydrogenase, and adenosine triphosphatase. Zejli [19] assessed the efficacy of extracts collected from Origanum grossii and *Thymus pallidus* leaves. Those plants are full of phenolic compounds, which always show high antioxidant activities. MeOH, H_2_O, and ethyl acetate were applied as extractants during the process of hot extraction through a Soxhlet apparatus. The MeOH extracts displayed the greatest reducing (0.101 and 0.188 mg/mL) and antiradical (0.067 and 0.153 mg/mL) powers, along with the highest total antioxidant capacities (TAC), for both plant species. To be clear, the TAC was based on mg AAE/g DW as a unit. In this unit, AAE means ascorbic acid equivalents and DW means dried weight. Mohammed [20] studied the chemical composition of *Adansonia digitata* L. fruit pulp, which is important because of its medicinal values [21]. A total of 18 phenolics and flavonoids were isolated, and the DPPH of leaf MeOH extracts demonstrated the potential of A. digitata leaves as a promising source of natural antioxidant compounds.

The natural extracts mentioned above with preservative properties are summarized in Table 1 and Table 2.

## 3. Preservation Materials Containing a Single Active Compound

Wang [22] embedded mycosporine-like amino acids (MAAs extracted from dried Pyropia haitanensis) in fish gelatin (FG) and oxidized starch (OS) and prepared active film (FOM film). Under light, the PV, acid value (AV), and free radical scavenging rate of oil samples wrapped in FOM were higher than those of samples without FOM wrapping. The composite film had excellent UV resistance, which was beneficial for extending the shelf life of grease. The effect of FOM film on the preservation of winter dates without lids was also determined. According to the residues of VC in jujubes, the contents in covered samples were higher than those in controls.

Chitosan has good film-forming properties, self-antibacterial properties [23,24], and self-antioxidant properties [25]. Bian [26] grafted gallic acid (GA) onto N-carboxymethyl chitosan (N-CMCS) by chemical bonding to form a freshness-retaining coating. This coating was applied on the surfaces of strawberries. The 1.5% GA-g-CMCS coating helped to not only prevent the weight and nutrients (soluble solid content (SSC), titratable acidity (TA), and ascorbic acid (AsA)) from being lost quickly but also maintained the activity of antioxidase.

Liu [27] prepared five phenolic acid–chitosan composite films and determined their preservation capabilities through weight loss rate (WLR), pH value, total volatile base nitrogen (TVB-N), thiobarbituric acid value (TBA), total bacterial count (TBC), and sensory score during storage of shrimp. Five phenolic acids were utilized, and they were *p*-coumaric acid, ferulic acid, vanillic acid, gallic acid, and salicylic acid. Among all composite films, GA–CS films possessed the best comprehensive properties, probably because of the three phenolic hydroxyl groups in their molecules. The FA–CS films showed better preservative properties on the shrimp, partly due to the methoxy group in ferulic acid.

In order to enhance the broad-spectrum light barrier and antimicrobial activity, Orsuwan [28] prepared a novel photosensitizing LDPE film containing riboflavin (vitamin B2, RB). Plants oils contain a high amount of chlorophyll, so their photooxidative stability is generally poor. Owing to excellent light absorption in the UV–Vis regions (approximately 200–500 nm), LDPE-RB composite films were studied for extra virgin olive oil (EVOO) to preserve essential nutrients. The results showed that the composite films effectively retained the necessary pigments chlorophyll and β-Carotene and prolonged the quality of the food.

Wang [29] prepared carvacrol-loaded PLA antimicrobial films and tested the influence of food microstructures on the release behavior of volatile antimicrobials. A food gel model structured to imitate jellies, jams, and dressings was utilized. The mechanical strength and water-retaining capacity were inversely proportional to the concentration of NaCl. The results demonstrated that the microstructures of the gels had a clear effect on carvacrol absorption, and this factor should be taken into account when designing antimicrobial packaging for the preservation of gel-like foods.

Zhang [30] developed procyanidin–chitosan composite films (PC–CS). Compared with PC-free CS films as controls, the antimicrobial properties of PC–CS films against *E. coli* and *A. niger* increased by 20.0% and 30.4%, respectively. The scavenging rate of DPPH and ABTS+ improved 2.45 times. The films also exhibited an interesting visualization detection capacity. In detail, pH responsivity was represented by outstanding changes in color.

Liu [31] prepared gluten- and glutenin-based films containing polyphenols; i.e., polyphenols named naringin (Na), cyanidin-3-O-glucoside (Cg), and proanthocyanidin (Pr). Compared with gluten-based films, glutenin-based films exhibited better antioxidant properties according to a free radical scavenging assay. This may be due to the high hydrophilicity of the glutenin, which increased the release rate of phenolic substances from the films to the ABTS system. All films except Cg films inhibited the growth of *E. coli*.

Prakash [32] prepared ALG films containing citral nanoemulsion and determined the preservative properties of fresh-cut pineapples. The films were edible and helpful in prolonging the shelf life of fresh-cut pineapples. Further exploration of 0.5% citral nanoemulsion could be useful for commercial applications.

## 4. Preservation Materials Containing Mixed Extracts

### 4.1. Essential Oil

Plant essential oil is a mixture extracted from the flowers, leaves, roots, bark, fruits, seeds, and other parts of plants through distillation or squeezing. Plant essential oils are generally composed of over a hundred components, including phenols, acids, alcohols, aldehydes, ketones, ethers, lactones, oxides, and terpenes.

Plant essential oils can purify the air, and their pleasant aroma can relieve fatigue [33] and improve sleep [34]. They can also be anti-inflammatory [35], antioxidant, and bactericidal, with medicinal values [36].

Essential oils are recommended by many researchers. There is an unknown synergistic antibacterial effect between different natural extracts, and essential oils themselves are mixtures of multiple active substances.

According to the Food and Drug Administration (FDA) of the United States, plant essential oils are generally classified as certified GRAS (Generally Recognized as Safe) substances. GRAS substances and food additives (FA) are two different regulatory identities for food ingredients besides new dietary ingredients (NDI). The Federal Food, Drug, and Cosmetic Act (FD&C Act), published on the official website [37], explains that GRAS is a regulatory classification term used to identify “food additives that are generally recognized by qualified experts as safe under their intended conditions of use.”

Considering the instability caused by the volatility of essential oils, the odor stimulation caused by high concentrations, and the skin and mucous irritation caused by high phenolic contents, a series of problems such as damaging consumer sensory experience can occur. Wrapping essential oils in food-grade materials before adding them to a film-forming matrix is an effective improvement.

Nurain [38] collected ethanolic extract and essential oil from *Persicaria hydropiper* and then added them to PLA to prepare antibacterial packaging. Based on the disc diffusion assay, both the ethanolic extract and essential oil of *P. hydropiper* exhibited antibacterial activity against bacteria *S. aureus* 6538P. The *P. hydropiper* ethanolic extract and essential oil also exhibited antibacterial activity in PLA film against *S. aureus* 6538P. The results of the antibacterial activity of the PLA films showed that the films had potential for antimicrobial packaging.

Shi [39], with the help of electrospinning technology, firstly encapsulated oregano essential oil (OEO) into β-cyclodextrin (β-CD) to prepare nanofibers and secondly fabricated fibrous films with PLA and PCL for fruit packaging. The films were called OEO@β-CDs/PLA/PCL. The nanofibers had not only preservation effects but also biosafety. The films delayed deterioration, postharvest decay, and storage quality loss in blackberries. The deterioration of decay rates, weight loss, firmness, TSS, and the appearance of fruits with OEO@β-CDs/PLA/PCL treatment were the lowest compared with other groups. In addition, nanofibers co-cultured with zebrafish for 15 days maintained a high vitality.

Dogan [40] fabricated novel active cheese packaging using lemon peel oil and gelatin. The main chemical composition of the lemon peel oil was limonene (60.4%). A total of 16 components were identified, which indicated that lemon peel had many unknown benefits. Gelatin fibrous mats loaded with lemon peel oil were fabricated through centrifugal spinning and then crosslinked. The crosslinked gelatin fibrous mats positively affected the shelf life of cheese.

Li [41] added garlic essential oil and anthocyanins extracted from purple cabbage to water-soluble modified chitosan (GCS) and gelatin polyelectrolyte complex to form films with antioxidant activity and antibacterial activity. After it had been grafted with gallic acid, chitosan was ingeniously utilized as not only a film substrate but also an emulsifier. Two films were fabricated and compared. A composite film called GF/GCS was fabricated with gelatin and GCS. The other film was fabricated with the composite film and garlic essential oil. Anthocyanins were utilized as pH-responsive smart labels in order to endow the film with visual detection activity. Compared with commercial PE film, the films displayed better preservation effects on cherry tomatoes and fish, which was shown by the smaller decrease in total phenolic contents and titratable acids. Both films and smart labels were confirmed as non-toxic.

Using casting and solvent evaporation, Yi [42] prepared active biodegradable films consisting of chitosan (CS) and PVA containing 0.125%–1% *w*/*w Carica papaya* seed essential oil (CPEO). The films were applied to prevent lipid food oxidation, proving their effective shield against UV light. The testing also demonstrated that the composite film possessed promising cytocompatibility with two human normal cells but high cytotoxicity against four human tumor cells.

In addition to direct addiction of the essential oils into the film-forming matrix, emulsion packaging technology can also be used, that is, the essential oil is first wrapped then stabilized and blended into the matrix material. An A-grade Pickering emulsion is mainly an emulsion stabilized by polysaccharides, proteins, or their composite particles, rather than traditional small molecule surfactants [43]. For food applications, nanoemulsion technology allows the incorporation of hydrophilic and lipophilic substances with antibacterial and antioxidant properties, which can be released during storage to extend the shelf life of various products [44]. The properties and stability of nano lotion depend on the preparation method, the addition order of raw materials, and the phase change during the emulsification process.

Zhao [45] prepared Pickering nanoemulsions for cinnamon-perilla essential oil (C-PEO) and collagen (as an emulsifier) and then imparted nanoemulsions into chitosan to prepare edible films. These films were investigated by preventing chilled fish fillets from significant quality declines. During 8 days of storage, the samples wrapped by these films showed lower TVB-N and Thiobarbituric acid (TBAR) values compared with those samples without films. The TVB-N content of the control was beyond 30 mg/100 g (the acceptable limit) only after the 6th day of storage; while the samples wrapped with active films showed the best results, the value outran the limit value after the 12th day. A synergistic effect between plant essential oil Pickering emulsion and anthocyanidin was recovered. Hao [46] developed ALG films containing an emulsion of thyme, pimento, and oregano essential oils, which were utilized for preserving fish fillets at two different temperatures, 4 and 20 °C. EO-emulsion-based ALG coating showed good antibacterial activity with a high-value sensory assessment. The Enterobacteriaceae (ENT) level is an important standard for evaluating the hygiene status of refrigerated food. The ENT levels of three groups, fillets without coating (CK−), fillets with EO-free-coating (CK+), and fillets with EO-coating, were tested. After day 8, the ENT levels of P, O, and T were significantly lower than those of CK− and CK+, indicating that coating with the three EO-emulsions slowed the growth of ENT in carp fillets. The TVB-N of the EO-coating film groups maintained values constantly lower than 25 mg/100 g during storage. If the 1% EO emulsion coating could be removed before purchase, the fish fillets were acceptable for consumers.

Xiong [47] prepared an edible pectin (PEC) coating with oregano essential oil (OEO) and resveratrol (RES) nanoemulsion for keeping pork loin fresh. It was found that the edible emulsion coating not only significantly inhibited the growth and reproduction of microorganisms in pork loin but also maintained the tenderness of the pork.

Wang [48] encapsulated vanilla essential oil into octenyl succinic acid starch (OSA-starch) to prepare Pickering emulsion. Then, the antioxidant activity of the emulsion was tested. At the same oil content, the antioxidant activity of emulsion was better than that of pure oil. After 24 h storage, the antioxidant activity of the emulsion was enhanced, and the vanilla essential oil was slowly released from the emulsion.

Arellano [49] studied the inhibitory effects of a microemulsion composed of oregano oil, cinnamon oil, lemon grass oil, and plant-based emulsifier. *E. coli* O157: H7 and *P. fluorescens* were test strains. They were inoculated on the leaves of iceberg lettuce stored at 4 °C. The results indicate that 0.5% cinnamon and 0.3% oregano oil have the potential to become natural, environmentally friendly, and effective alternatives to chemical preservatives for extending the freshness of green leafy vegetables. The experimental group treated with 0.5% lemon grass and 0.3% oregano microemulsion significantly reduced the number of *E coli*. It only took 3 days to kill all bacteria (*p* < 0.05).

Pickering emulsion does not contain emulsifiers (which is different from nanoemulsions and microemulsions), but it uses solid particles as stabilizers. Microemulsion is a liquid system that differs from nanoemulsion, studied as early as 1959 by Schulman, which has thermodynamic stability and isotropic properties and can be spontaneously formed. A comparison of these emulsions is shown in Table 3.

Wei [52] prepared pullulan- and sodium alginate (PS)-based films with a thyme essential oil microemulsion. The films were utilized for preserving chilled pork. After 10 days of storage at 4 °C, the total viable count (TVC) of the chilled pork preserved in the PS-essential oil microemulsion material was significantly reduced compared with the control group. Feng [53] prepared glutenin and tamarind gum films using a melatonin/pummelo essential oil binary microemulsion. The films were utilized for preserving *Agaricus bisporus*. The effective attachment of the melatonin and essential oil layer in the films enhanced antioxidation, micro-organism inhibition, and free-radical-scavenging properties, which effectively delayed the senescence of post-harvest white mushrooms. Tavakoli [54] applied Water2/Oil/Water1 double emulsion (DEs) to prepare a delivery system for Spirulina platensis extract and Epsilon-poly-l-lysine. This system was capsuled by soybean polysaccharide (SBP)-bovine skin gelatin (GL) and then it was applied as a biodegradable preservation material for prolonging the freshness of fish. Pycia [55] added hazelnut oil microemulsion to furcellaran film in order to preserve cod liver oil. After 3 months of storage at 3 °C with access to light, the acid (AV), iodine (IV), and peroxide values (PV), together with a profile of the fatty acids, were determined for the oil samples. Lipid hydrolysis increased with higher AV values, resulting in an unpleasant taste and smell. The IV and clearly indicated the process of fat rancidity. In detail, IV is a measure of unsaturated fatty acid content, and PV is a measure of peroxide and hydroperoxide content. It was shown that the oil samples had a similar acid number. In addition, the IV and PV values significantly increased.

Laorenza [56] prepared biodegradable preservation materials for shrimp using poly (butyleneadipate-co-terephthalate) (PBAT)/PLA as a matrix and ginger oil (GO) and lime peel oil (LPO) as preservation agents. Essential oil loading led to improved film flexibility but reduced tensile strength. Films containing LPO were more effective in inhibiting microbial growth. Films containing GO showed superior prevention of melanosis in packaged shrimp.

### 4.2. Non Essential Oils

Using solution-casting methods, Ali Amjad [57] prepared corn starch-based antimicrobial and edible films. Three medicinal plants, acontium heterophyllum, artemisia annua, and thymus serpyllum, were used as fillers to reinforce the gelatinized solution. The films exhibited significant antioxidant potential and antibacterial activity. The corn starch-based films were not only antimicrobial and edible but also completely biodegradable because all of their ingredients came from natural food resources. In a word, these materials were safe as food packaging and were suitable for medicinal capsules as well.

Khalil [58] collected three bioactive ingredients from citrus peels; i.e., grapefruit peel methanolic extract (GFPE), grapefruit pectin (GFPec), and lemon peel extracts (LPE). First, LPE was encapsulated by maltodextrin (MD-LPE); second, active films named GFPec-GFPE/MD-LPE were prepared using MD-LPE, GFPec, and GFPE. The fabricated novel edible GFPec-based films are candidates for antimicrobial food packaging and shelf-life extension of fresh-cut produce, and they are considered promising eco-friendly alternatives to synthetic food packaging materials.

Liu [59] prepared composite films using chia seed mucilage, chitosan, and Xanthoceras sorbifolium leaf extract (CSM/CS/X). CSM/CS/X films with good biodegradability, antioxidant, and antibacterial activities are promising for packaging applications. According to dose-response relationship assays, the CSM/CS/X4 containing 4 wt.% leaf extract showed relatively better performance, such as a smooth and homogenized surface, good tensile strength (22.28 MPa), elongation at break (11.86%), and highest decomposition temperature (202.6 °C). The results indicated that biowaste X. sorbifolium leaf could be utilized to fabricate eco-friendly packaging films, which are expected to replace petroleum-based packaging materials.

Madureira [60] prepared biodegradable PLA and oriented polypropylene (OPP) with natural extracts of olive pomace (EXT) for prolonging the quality and freshness of fresh-cut apples. Commercial ascorbic acid was applied as a control. After refrigerating for 12 days, the total phenolic index and antioxidant potential of the fruit were preserved without a significant decrease in firmness. Through evaluating CO_2_ production, the respiration rate was assessed as moderate, and no detection of coliforms was verified throughout the 12 days of storage. According to microbial load studies, two points were worthy of notice. First, EXT showed benefits in inhibiting three kinds of microbes’ growth in apple slices; second, PLA showed higher inhibition than OPP. The overall results demonstrated that PLA with EXT could be applied as a preservation film for fruits at refrigeration temperature for 5 days. The recommended limits of the microbial load of tested microbes and the microbial load on PLA- and OPP-packaged fresh-cut apples after 12 days of storage at 4 °C are shown in Table 4.

Andrade [61] prepared PLA active food packaging films with three natural extracts, including grape and pomegranate by-products. Moreover, the effectiveness of the new film was carried out through the study of the lipid oxidation state and microbial contamination of two high-fat content foodstuffs, almonds and beef. As for evaluating vitro antioxidant activities, three extracts were applied. They were wort extract, freeze-dried pomegranate peel extract (PPE-FD), and natural pomegranate peel (PPE-N). PPE-FD presented the highest inhibition percentage (IP = 175.3 ± 0.38 mg TE/g) and content of total phenolic compounds (TPC = 221.5 ± 0.62 mg GAE/g) and total flavonoids (TFC = 31.39 ± 0.61 mg ECE/g). As for active films, PLA incorporated with 3% pomegranate peel (PLA/3PP) showed better antioxidant activities than PLA incorporated with 3% pomegranate extract (PLA/3PPE). Both active films showed antimicrobial activity against *S. aureus*. Only PLA/3PPE seemed to indicate potential antimicrobial activity against *L. monocytogenes*. Neither PLA/3PP nor PLA/3PPE showed antimicrobial activity against *E. coli* and *E. faecalis*. The results from food assays showed that delays in the lipid oxidation of active PLA films were not effective in almonds but significantly effective in beef. The microbial growth in beef was reduced over time.

Fan [62] prepared chitosan-starch film with Portulaca oleracea extract (POE), a kind of natural extract, to investigate the potential application of preserving chilled meat. Aside from CS incorporated with 0.15% of an extract called CS/POE (0.45%), other active films showed lower TBV-N values than the threshold limit (15 mg/100 g). CS/POE films had a good color-protecting effect in suppressing appearance changes (L*, a*, b*, ΔE) in the meat and remarkable antioxidant activity, and they significantly reduced lipid oxidation in the meat.

To preserve oxygen-sensitive foodstuffs, Stoll [63] utilized bixin and PLA to fabricate active packaging for sunflower oil. Bixin was extracted from annatto seeds. After exposure to intense light for 350 h, the solutions covered by film produced by melt processing with bixin (MP.Bix) and plasticized film produced by melt processing with bixin (MP.Bix.P) preserved nearly 80% of the riboflavin. In the five-day accelerated storage, the active films kept the peroxide level under the limit of commercialization (10 mEq/kg). In order to measure the migration rate of bixin, a food simulant of fatty foods consisting of 95 vol.% ethanol and two processing methods cast together with melt were applied for 16 days at 40 °C. The initial bixin release from the MP was 3-fold faster than that from the Cast.Bix. The faster release of bixin to 95 vol.% ethanol in the MP films might be related to PLA chain thermo-hydrolysis [64] or thermo-mechanical degradation during the melting process at high temperatures, the presence of oxygen, or shear. These phenomena may facilitate bixin diffusion through the polymeric matrix.

In order to utilize cardoon oil cake proteins (CPs) to make preservation sachets for peanuts, Mirpoor [65] crosslinked CPs using an enzyme named microbial transglutaminase (mTGase). It was the first time CPs and mTGase were combined. According to a bio-disintegration test, all films were broken into pieces after 1 month; after 50 days, no matter the concentration of mTGase, more than 80% of the films were degraded. Two parameters—peroxide value (PV) and water content (WC)—were applied as measures to characterize the quality of peanuts during storage. When the value of PV reaches 10 meq/kg oil, generally speaking, the food is judged to be at the end of its shelf life [66]. G. Rossi-Márquez’s work [67] showed that the water content of peanuts should not exceed 2.9%. It was interesting that samples wrapped with LDPE remained below this value for 28 days, while the samples wrapped with CP films only remained below the value for 15–20 days. From a macro perspective, films with more mTGase-catalyzed extracts showed higher contact angles, which indicated that the enzyme could cause intermolecular isopeptide bonds between the CP chains, resulting in a more compact and continuous protein matrix. SEM images from a micro perspective evidenced this. In a word, this novel and biodegradable packaging could be an effective tool to mitigate peroxide production together with water content loss.

In order to improve the shelf quantity of grapes, Karkar [68] fabricated edible packaging using chitosan (CS) and *Nigella sativa* (NS) extracts, which was called NS-loaded-CS film. Natural ethanolic (NS-Et) and methanolic (NS-Met) extracts were collected and then added to the chitosan. These two active films are named Et-CS and Met-CS for convenience in this review, thereby differing from the original authors’ nomenclature. According to gastrointestinal digestion, antioxidant capacity increased more than 15-fold. Physical changes in grapes with active films were examined, such as color, volume, and mildew. The results showed the grapes covered with films were fresh and smooth, while uncovered grapes were not.

## 5. Food Safety of Natural Antibacterial Extracts

Common toxic crosslinking agents generally cannot be utilized in food packaging. Cinnamaldehyde, as a natural crosslinking agent, is an effective substitute. Balaguer [69] prepared crosslinked films using cinnamaldehyde and wheat gliadin. Because of crosslinking, the mechanical and barrier properties (O_2_, CO_2_, H_2_O) of the films were enhanced, while the degradability was not affected.

Zahra [70] prepared ALG coatings containing citrus and lemon extracts (CAE and CLE, respectively) and applied them to chicken stored at 4 °C for 16 days. The coating was edible, antioxidant, and antimicrobial. First, significant positive effects on the values of pH, TVB-N, PV, and TBA (*p* < 0.05) were found, which contributed to the quality of the chicken. Second, the lowest microbial counts were observed in ALG-CAE-CLE coating at an extract concentration of 2%. From a sensory perspective, in other words, taste, odor, color, and texture, good overall acceptability scores were shown in coated samples. In a word, meat coatings could take advantage of extracts from citrus fruits.

Praveenkumar [71] investigated the effect of extracts from lemon peels (LPE) and/or pomelo (PPE) combined with copper sulfide nanoparticles (CuSNPs) on Melanosis of Indian White Prawn in cold storage for 15 days. Biochemical indicators of the treated prawns, such as trimethylamine (TMA), TVB-N, FFA, and PV, microorganism counts, such as aerobic bacterial count (ABC), and melanosis score, all decreased, while the sensory scores were higher than those of the untreated sample. Biopreservation techniques may not only provide a new alternative to preservation techniques but may also impact shelf life and product safety.

Yaghoubi [72] evaluated the physico-chemical, microbiological, and sensory changes caused by nanoparticle (NPs) packaging with oleaster leaf essential oils (OLEOs) in emulsion-type sausages without added chemical nitrite/nitrate salts at 4 °C for 45 days. Two NPs were researched in the investigation; i.e., nisin nanoparticles (NI-NPs) and ε-polylysine nanoparticles (ε-PL-NPs). The use of combined e-PL-NPs with Ni-NPs with OLEOs resulted in a higher pH value, TPC, and lower TBARS, and also a decreased total viable pathogen (TVP). The sensory traits of the newly formulated sausages were basically approved by consumers.

All the preservative films/coatings/emulsions containing natural extracts mentioned above are summarized in Table 5 and Table 6.

## 6. Conclusions and Prospects

In order to expand shelf life and to detect and identify freshness, deoxidizers, antimicrobial placers, biosensors, or other active components are put into packaging. This new kind of packaging is defined as active packaging.

In the food industry, active packaging can play a role in preventing food pollution and maintaining freshness and nutrients. As we all know, mining for petroleum could seriously damage the environment and ecology. In addition, petroleum resources are nonrenewable without a very long wait. Fortunately, abundant natural resources lay the foundation for active packaging with natural antimicrobial extracts. Biobased polymers have excellent properties such as biodegradability, environmental friendliness, and non-toxicity, making them a novel substitute for traditional petroleum-based packaging materials. Supercritical extraction does not damage the components of essential oils, but the cost is too high and the operation is difficult.

Natural plant extracts are not inferior to chemical synthetic antibacterial agents in terms of antibacterial properties and freshness retention. Because the extracts are mixtures, they often show extensive inhibition against a variety of fungi, bacteria, and viruses, and many components have synergistic effects themselves, which can be directly combined using a variety of refined oils.

Although research on active packaging containing natural extracts continues to emerge, there are still many issues to be explored. Five suggestions are proposed below:

Extraction technology utilized in plants’ active components. The most commonly utilized technologies currently include distillation, squeezing, chemical solvent extraction, and CO_2_ supercritical extraction, etc. Distillation is the most traditional method for extracting essential oils, but some drawbacks are inevitable; e.g., being time-consuming, low yield, and vitiating certain components. Chemical solvents generally have a pungent odor. For example, alcohols inhaled into the human body can cause mucosal and neurological damage, and alcohols are highly flammable liquids with certain risks during usage.

The good news is that new technologies are currently emerging, such as the biological enzymatic hydrolysis-assisted distillation method, ultrasound combined with the enzyme-assisted distillation method, cellulase-assisted Soxhlet extraction, the microwave ultrasound method, the solventless microwave method, and the low-temperature continuous phase transformation method, etc. Pulsed light and pulsed magnetic fields are also expected to be combined with lotion technology so that the greatest extent of active components can be retained, which will be conducive to the better utilization of antioxidant properties.

Further research on the mechanisms of antimicrobial activity is needed.

Controlled and slow release. Nanotechnology can achieve sustained release of drugs, but the dynamics of release are not thorough enough. Controlled release is more effective in achieving efficient drug utilization than sustained release.

More preparation technologies for materials should be introduced into active packaging, such as 3D printing technology, which is suitable for personalized packaging. Owing to personalized packaging being adopted for different foods, consumers would undoubtedly prefer it.

Intelligent packaging with indicator functions. Some substances may exhibit different colors at different temperatures or pH values, which not only inhibits bacteria but also visualizes the freshness of the food.

## Figures and Tables

**Table 1:** Antioxidant properties of natural extracts.

Source	Chemical Compounds	Antioxidant Experiments			Ref.
		Experiment	Results		
*Inula grandiflora*	10 phenols (vanillic acid, vanillin, ferulic acid, etc.) and 5 flavonoids	DPPH/ (µg/mL)	IC_50_ = 55.13 ± 1.84 − 442.8 ± 12.13		[10]
*origanum grossii* and *Thymus pallidus*	Naringin, Hesperidin, licoflavone C	TAC/ (mg AAE/g DW)	945.43 ± 7.98 (oregano)	928.407 ± 4.41 (thyme)	[19]
*Adansonia digitata* L.	Rutin (31.9), quercetin-3-β-d-glucoside (8.86), caffeic acid (5.33), etc.	DPPH/ (mg/mL)	IC_50_ = 0.23 ± 0.01		[20]
Citrus	-	*Salmonella* sterilization on cucumbers	Reduced by 1.8 (10 °C) and 2.5 (22 °C) logCFU/cm^2^		[17]

- means unknown, and similarly hereinafter.

**Table 2:** Antibacterial properties of natural extracts.

Source	Chemical Compounds	Microbial Species	Index	Mechanism	Ref.
-	Vanillin and cinnamic acid	4 food spoilage yeasts	MIC ≤ 0.125 mg/mL	The adherence on abiotic surface decreased.	[8]
-	Eugenol	*A. Carbonarius*	MIC = 0.8 μL/mL	The clustered genes for OTA biosynthesis were significantly reduced.	[9]
-	Isomers of β-citronellol	*C. albicans* *C. tropicalis*	MIC_50%_ = 64 µg/mL MIC_50%_ = 256 µg/mL	Both substances displayed aneffect on the fungal membrane but not on the fungal cell wall.	[11]
Ginger	6-gingerol, 6-shogaol, zingerone	*B. Subtilis* *P. aeruginosabacterium*	Biofilm activity: 50% subcritical water extract = 0.5% peracetic acid	Curcumene, 6-shogaol, and zingerone in ginger’s subcritical water extract, which destroyed biofilms.	[14]
-	PAE	*A. flavus*	The percentage of early apoptotic cells: (1) 27.4%(0.25 µg/mL PAE) (2) 48.7%(0.5 µg/mL PAE)	PAE induces fungal apoptosis through a caspase-dependent mitochondrial pathway.	[15]
POEO	Phytol, phytone, *n*-pentacosane, 1-octen-3-ol, and β-ionone	Pcc	MIC = 0.625 mg/mL	POEO destroyed cell morphology.	[18]

**Table 3:** Comparison of Pickering emulsion, nanoemulsion, and microemulsion.

	Nanoemulsion	Microemulsion	Pickering Emulsion
Composition	Water, oil, emulsifier	Water, oil, surfactant, cosurfactant	Water, oil, solid particles
Particle size	0.1–1 μm; monodispersed system	10–100 nm; monodisperse system, sphericity	<500 nm; nonsphericity [50,51] or sphericity
Optical property	Transparent or semi transparent	Transparent or semi transparent	Opaque
Stability	Dynamic stability	Thermodynamic stability	Dynamic stability
Spontaneous formation	No	Yes	Yes

**Table 4:** Microbial load on PLA- and OPP-packaged fresh-cut apples after storage (log CFU∙g^−1^).

	Mesophilic Bacteria	Filamentous Fungi	Coliforms
Recommended limits	6	2.7	4
PLA with EXT	3.75	3.3	not detected
OPP with EXT	5.2	5.25	not detected

**Table 5:** Preservative film/coating/emulsion-containing natural extracts for food.

Matrix Materials	Antibacterial Composition	Food Category	Storage Conditions	Index	Ref.
Pea protein isolate, candelilla wax	d-limonene	Tomato	Soaking treatment, 8 days	MIC = 12.5 mg/mL	[12]
Fish gelatin, oxidized starch	MAAs from dried Pyropia haitanensis	Grease and winter dates	Natural light, 2 days	PV, AV, and DPPH: higher *E. coli* survival rate: the decrease slowed down	[22]
N-CMCS	GA	Strawberry	20 ± 2 °C, RH 50%, 4 days	Decay rate = 36.7%–8.9% WLP = 12.7%–8.4% TA, AsA, and SSC residues: higher DPPH: better. Antioxidant enzyme activity: maintained	[26]
Sodium alginate	Citral nanoemulsion	Fresh-cut pineapple	37 °C, 12 days	0.5% citral nanoemulsion coated pineapple caused reduction of artificially inoculated food-borne pathogens and were sensory accepted.	[32]
PAL and PCL	OEO	Blackberry	Dark, 4 ± 1 °C, 90% RH, 4 days	Decay rate: less than 50% Weight loss: less than 15% TSS decreased by 15%	[39]
Chitosan and gelatin	Garlic essential oil and anthocyanins from purple cabbage	Cherry tomato/fish	room temperature for 9 days/4 °C for 3 days.	TVB-N (fish): ~15 mg/100 mg Weight loss (fruit): 4% Titratable acid content and total phenolic content(fruit): higher	[41]
Grapefruit pectin (GFPec)	MD-LPE, GFPec, and GFPE	Cherry tomato	Chilled, 6 days	Growth of *E. coli* O157:H7 inhibited by similar to 1.6 log units.	[58]
PLA and OPP	Olive pomace extracts	Apple	4 °C, 12 days	Respiration rate: 11 mmol CO_2_/kg·h, moderate Water vapor permeability: 1.10 × 10^−12^ (PLA) > 8.75 × 10^−14^ (OPP) mol·m/m^2^·s·Pa	[60]
Chitosan	Nigella sativa	Grape	1 week	Antioxidant capacities: (1) 255.85 (Et-CS) (2) 293.72 (Met-CS) Grape coating studies. (1) no obvious changes on the surface (Et-CS) (2) from 4th day, blacking and deformation (Met-CS)	[68]
LDPE	Vitamin B2	EVOO	Ultraviolet and short-visible light	Essential pigments preserved. Antibacterial activity: reduced by >99%(Gram-negative) and 94% (Gram-positive)	[28]
A plant-based emulsifier	Oregano oil or lemon grass oil or cinnamon oil	Iceberg lettuce	4 °C 28 days	Survivors: all lower; No surviving populations by day 3	[49]
Glutenin and tamarind gum	Melatonin/ pommelo essential oil	White mushroom	3 ± 1 °C, 12 days	Respiration rate: 400–600 nmol kg^−1^ s^−1^ MDA: 0.7–1.3 mmol kg^−1^	[53]
Furcellaran	Hazelnut oil Microemulsion	Cod liver oil	22 ± 1 °C, 3 months	AV: similar IV: a significant decrease of 47% PV: 4, 17 and 27 times	[55]
PLA	Wort, grape, Pomegranate	Almond Beef	(1) 40 ± 1 °C, 30 days (2) 23 °C, 21 days 4 °C, 11 days	IP of films: 7.19 and 13.34% (1) Almonds: no significant differences (2) Beef: lower malonaldehyde equivalents	[61]
PLA	Bixin	Sunflower oil	40 °C, light or dark, 15 days	Residue of riboflavin: ~80% Peroxides level: <10 meq/kg for 5 days	[63]
Cps	mTGase	Peanut	33 ± 2.5 °C, the RH 65 ± 5%, 28 days	Residue of riboflavin: ~80% Peroxides level: <10 meq/kg for 5 days	[65]
Gelatin	Lemon peel oil	Cheese	4 °C, 28 days	Microbial counts decreased 2.3 (*S. aureus*) and 2.04 (*E. coli*) logs	[40]
Chitosan	C-PEO Pickering nanoemulsions	Red sea bream fillets	Chilled, 14 days	DPPH: 65–80% TVB-N < 30 mg/100 g for 12 days (longest) TBA < 0.20 < 0.35 (control) mg MDA/kg	[45]
Sodium alginate	Thyme, oregano, and pimento essential oil emulsion	Chilled carp fillets	10 days	ENT: significantly lower than CK− and CK+ after 8 days TBV-N: significantly lower than CK− and CK+ after 6 days	[46]
Pectin	OEO and RES	Pork loin	4 °C, 20 days	Lipid oxidation greatly retarded. Protein oxidation less severe. Sensory quality: the shear force values of all groups were initially decreased, reaching the lowest level on days 5–10, followed by a rapid increase thereafter. TVC: significantly reduced	[47]
Pullulan and sodium alginate	Thyme essential oil microemulsion	Pork	4 °C, 10 days	Antioxidant activities: significantly better TVC: significantly reduced	[52]
Soybean polysaccharide and bovine skin gelatin	S. platensis	Grass carp fillets	4 °C, 10 days	Overall acceptability: 3.82–5.12	[54]
PBAT/PLA	GO and LPO	Shrimp	4 °C, 6 days	TVC = 7 Log CFU/g	[56]
Chitosan and starch	Portulaca oleracea extract	Meat	Chilled, 16 days	DPPH: 83.67–182.33% TBARS analysis: decreased by 21.8% (PE) and 28.7% (CS) TVB-N: 14.89 (0.30%) and 14.57 (0.45%) < 15 mg/100 g	[62]
Sodium alginate	Citrus and lemon extracts	Chicken	4 °C, 16 days	The peroxide value (mEq/kg), the TBA value (mg MDA/kg), and the TVC (log10 CFU/g) all below the control during 16 days storage.	[70]
CuSNPs	LPE and/or PPE	Indian white shrimp	Chilled, 15 days	TMA = 10.94 ± 1.04 < 18.01 ± 0.79 mgN/100 g TVB-N = 17.51 ± 1.1 < 45.08 ± 1.1 mgN/100 g FFA = 0.091 ± 0.01 < 0.141 ± 0.001% PV = 7.57 ± 0.38 < 16.42 ± 0.61 milliequivalents/kg ABC = 6.66 ± 0.4 × 10^7^ < 2.166 ± 0.3 × 10^9^ Melanosis score = 60% < 80%	[71]
ε-PL and nisin nanoparticles	Oleaster leaf essential oil	Emulsion-type Sausages	Vacuum PE bag, 4 °C, 45 days	Total viable bacteria values all decreased (1.28 Log CFU/g): (1) 1.43 Log CFU/g for *Clostridium perfringens*; (2) 0.24 Log CFU/g for *E. coli*, (3) 0.63 Log CFU/g for *S. aureus*, (4) 0.86 Log CFU/g for molds and yeasts.	[72]

**Table 6:** Preservative materials containing natural extracts.

Matrix Materials	Antibacterial Composition	Food Category	Storage Conditions	Index	Ref.
Chia seed, chitosan	Xanthoceras sorbifolium leaf	4 food simulants	Normal, 60 min	The antioxidant capacities: up to 1–3 folds Inhibition zone = 3.55–7.82 (*S. aureus*) and 0.60–5.14 (*E. coli*)	[59]
PLA	Persicaria hydropiper	*S. aureus* 6538P	37 ± 2 °C, 24 h	MIC = 0.625 and 5 mg/mL MBC = 5 and 40 mg/mL	[38]
Chitosan and PVA	CPEO	*E. coli* and *S. aureus*	37 °C, 24 h	Size of the inhibition zone < 30 mm. Effectiveness for *S. aureus* better than for *E. coli*	[42]
OSA-starch Pickering emulsion	Vanilla essential oil (30.54% vanillin)	-	25 ± 0.5 °C, 14 days	DPPH and ABTS+: better	[48]
Corn starch	Acontium heterophyllum, Artemisia annua, and Thymus serpyllum	*S.aureus* and *Salmonella*	37 °C, 24 h	Percent antioxidant activity: 0.00–69%. Diameter of zone inhibition increased compared with corn starch (0.00).	[57]

## Data Availability

Not applicable.
